# What are the sociodemographic and gender determinants of non-fatal self-harm in older adult users and non-users of antidepressants? A national population-based study

**DOI:** 10.1186/s12889-020-08892-2

**Published:** 2020-06-16

**Authors:** Khedidja Hedna, Johan Fastbom, Ingmar Skoog, Gunnel Hensing, Margda Waern

**Affiliations:** 1 Center for Ageing and Health (Age Cap), Department of Psychiatry and Neurochemistry, Gothenburg University, SE-413 45 Gothenburg, Sweden; 2 Statistikkonsulterna Jostat & Mr Sample AB, Gothenburg, Sweden; 3 Aging Research Center, Karolinska Institutet and Stockholm University, Stockholm, Sweden; 4 Department of Psychiatry, Cognition and Old Age, Sahlgrenska University Hospital, Region Västra Götaland, Gothenburg, Sweden; 5 Section of Epidemiology and Social Medicine, Department of Public Health and Community Medicine at Institute of Medicine, University of Gothenburg, Gothenburg, Sweden; 6 Psychosis Clinic, Sahlgrenska University Hospital, Region Västra Götaland, Gothenburg, Sweden

**Keywords:** Pharmacoepidemiology, Sociodemographic factors, Self-harm, Cohort study, Older adults, Antidepressants, Registries

## Abstract

**Background:**

Late-life self-harm (SH) is often linked to depression. However, very few studies have explored the role of other factors and their interaction with depression in the occurrence of late-life SH. The objective of this research was to examine sociodemographic and gender factors associated with non-fatal SH, in older adults with and without antidepressant therapy.

**Methods:**

We used national longitudinal register data from a total cohort of all Swedish residents aged ≥75 years between 2006 and 2014 (*N* = 1,413,806). Using personal identity numbers, we linked individuals’ data from numerous national registers. We identified all those with at least one episode of non-fatal self-harm (regardless of level of intent to die) and matched 50 controls to each case. A nested case–control design was used to investigate sociodemographic factors associated with non-fatal SH in the total cohort and among antidepressant users and non-users. Risk factors were analysed in adjusted conditional logistic regression models for the entire cohort and by gender.

**Results:**

In all, 2242 individuals had at least one episode of a non-fatal SH (980 men and 1262 women). Being unmarried was a risk factor for non-fatal SH in men but not in women. Among users of antidepressants, higher non-fatal SH risk was observed in those born outside the Nordic countries (IRR: 1.44; 95% CI: 1.11–1.86), whereas in AD non-users increased risk was seen in those from Nordic countries other than Sweden (IRR: 1.58; 95% CI: 1.08–2.29). Antidepressant users with higher education had an increased risk of non-fatal SH (IRR: 1.34; 95% CI: 1.12–1.61), in both men and women.

**Conclusions:**

Foreign country of birth was associated with increased risk for non-fatal SH in older adults with and without AD therapies. Being married was a protective factor for non-fatal SH in men. The complex association between sociodemographic factors and use of antidepressants in the occurrence of self-harm in older men and women indicates the need for multifaceted tailored preventive strategies including healthcare and social services alike.

## Background

Non-fatal self-harm (SH) is a significant risk factor of subsequent suicidal behaviour in late-life [1]. Given the high rates of suicide in persons aged 70 and above, especially in men [2], and the high intent to die observed in older adult suicide attempters [3], exploring factors associated with non-fatal SH may help to inform interventions to prevent suicide. Research conducted in that field, however, tends to include a wide age range when defining older adulthood, with some including persons in their fifties [4, 5]. Further, many studies are hospital-based [1]. Since SH in late-life has distinct characteristics compared to younger populations [1], there is a need for research within a broader public health context, focusing on older adults in the general population.

Among adults who seek mental health care, the link between depression and suicide is particularly strong in those over the age of 75 [6], and most suicide prevention strategies have focused on the optimisation of the diagnosis and treatment of late-life depression [7]. However, it is critical to also explore the role of sociodemographic factors and their interaction with depression in the occurrence of late-life SH [1]. Further, very few studies have investigated the associated gender differences, and studies that focus specifically on those above the age of 75 are uncommon [8].

Large population-based studies provide a useful context and enough power to examine these factors without selection bias. We recently applied this approach to a study in adults aged 75 and above who were new users of antidepressant (AD) therapy [9]. We found an increased risk of non-fatal SH in women born outside of Nordic countries, but not men, and in those with higher education [9]. We therefore aimed to extend findings from our previous study and to explore the factors associated with non-fatal SH in the total population aged 75+, and with respect to treatment with AD.

## Methods

### Study population

Swedish residents aged ≥75 years (mean age 80.5 years; range 75–114) between January 1, 2007 and June 30, 2014 (*N* = 1,415,386) were included in a population-based register study and followed until December 31, 2014 or until date of emigration or death. About three out of five were women (58%), about half (47%) were married and nine out of 10 were born in Sweden (89%). Half of the population had compulsory school only (51%); a similar proportion (46%) had a blue-collar occupation before retirement. Nearly one-tenth (9%) resided in an institution.

A person was considered to be an AD user if a prescription for any non-tricyclic AD was redeemed for this individual during the study period according to the Swedish Prescribed Drug Register. We excluded those who used exclusively tricyclic AD, which are primarily prescribed for pain in Sweden today. The AD user cohort had higher proportions of women (67% vs 54% in non-users of AD), widows/widowers (43% vs 30% in non-users) and persons residing in institutions (21% vs 4% in non-users).

### Data sources

Data from national registers were merged through the unique personal identity number [10]. Users of AD were identified from the Swedish Prescribed Drug Register held at the National Board of Health and Welfare [11]. The register contains data with unique personal identifiers of all persons with redeemed prescriptions in the community, residential care and nursing homes. The National Patient Register, which includes all inpatient and specialised outpatient healthcare contacts, was used to identify individuals with non-fatal SH. Sociodemographic and emigration data were collected from Statistics Sweden. Persons residing in institutions were identified by the National Care and Social Services database. Death data were retrieved from the Cause of Death Register.

### Study outcomes

Persons with non-fatal SH (regardless of level of intent to die) were identified based on the International Classification of Disease (ICD)-10: Intentional SH (X60-X84), harm of undetermined intent (Y10-Y34), sequelae of intentional SH (Y87.0) and events of undetermined intent (Y87.2).

### Sociodemographic characteristics

Sociodemographic characteristics included: sex, age group (75–79, 80–84, 85–89, and ≥ 90 years), marital status (single, married, divorced, and widow/widower), country of birth (Sweden, other Nordic countries, and outside of Nordic countries), highest level of education (primary (compulsory), secondary, and higher education), category of employment at retirement (upper white-collar; lower white-collar; blue-collar and others), residence in institution at baseline. Annual disposable household income was categorised into quartiles.

### Statistical analysis

We first assessed the incidence rates of non-fatal SH in the total cohort and in users and non-users of AD. Then, employing a nested case-control design, persons with non-fatal SH were individually matched to 50 controls from the same age, sex, and time-specific risk set. For persons with more than one episode of non-fatal SH during the study period, we only considered as outcome the first episode that occurred during the study period. We generated conditional logistic regression models to estimate incidence rate ratios (IRR) in the total cohort and in AD users and non-users separately [12]. We fitted sex interaction terms and formally tested their significance. Analyses were then stratified by gender. We added to the adjusted regression models the following potential confounders: the concomitant use of other psychoactive medications, occurrence of non-fatal SH in the preceding year and use of specialised psychiatric care as a proxy for more serious mental disorder. The rationale for the latter is that milder forms of depression are treated within primary care in Sweden; more serious cases are referred to specialised care. The analyses were conducted using SAS statistical software version 9.4 (SAS Institute Inc., NC, USA).

## Results

In all, 2242 individuals (980 men and 1262 women) had at least one non-fatal SH episode during the study period (Table 1). The incidence rate of non-fatal SH among AD users was 178.6 per 100,000 person-years, a rate about 17 times that in the non-user group (10.5 per 100,000).

**Table 1:** Numbers and incidence rates of men and women with non-fatal self-harm in the total Swedish population aged ≥75 years and in antidepressants users and non-users

		N	Person-years	Persons with non-fatal self-harm (N)	Incidence rate of non-fatal self-harm per 100,000 person-year
**All 75+**	**All 75+**	1,413,806	6,106,791	2242	36.7
	**Men**	596,652	2,570,948	980	38.1
	**Women**	817,154	3,535,843	1262	35.7
**Non-users of AD**	**All 75+**	1,004,641	5,085,385	534	10.5
	**Men**	462,365	2,269,389	310	13.7
	**Women**	542,276	2,815,996	224	8
**Users of AD**	**All 75+**	373,661	826,390	1476	178.6
	**Men**	123,096	241,672	592	245
	**Women**	250,565	584,718	884	151.2

*N* Number; *AD* Antidepressants

### Total cohort

**Fig. 1:**
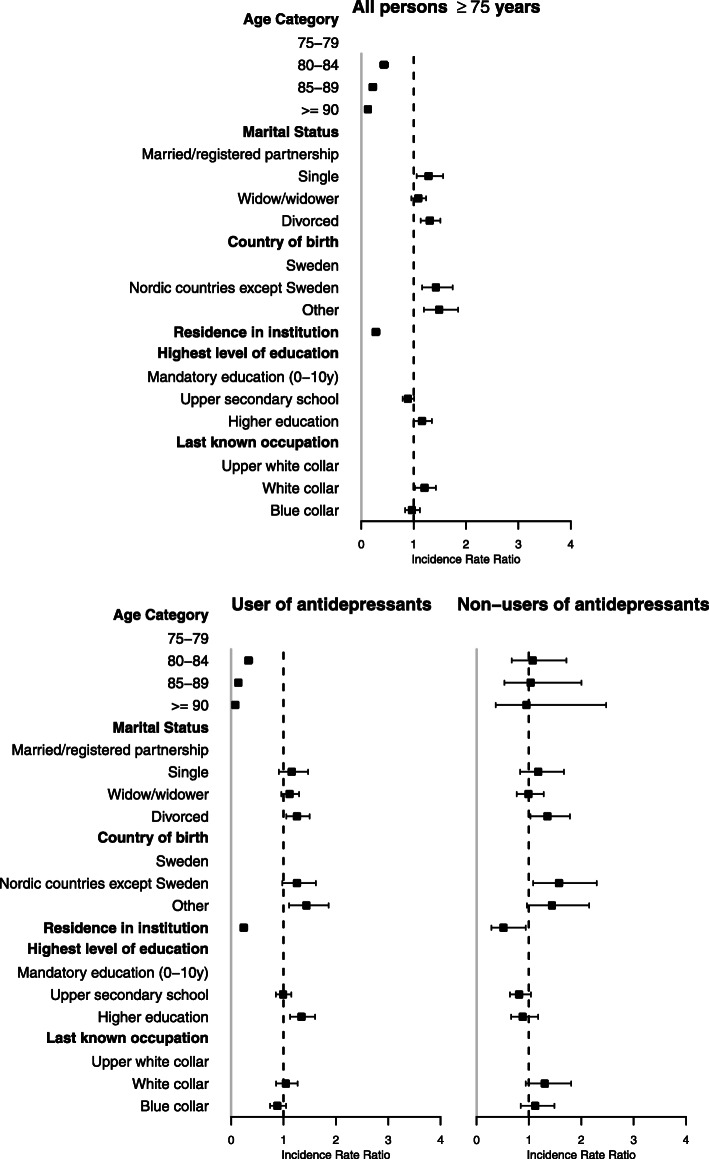
Factors associated with non-fatal self-harm in all persons aged ≥75 years and in antidepressants users and non-users. The regression models were adjusted for age, country of birth, marital status, highest level of education, last registered occupation, monthly individual disposable income, use of specialised psychiatric care, use of other psychoactive medications, residence in institution at baseline and non-fatal self-harm in the previous year

Being unmarried and being foreign born were associated with higher risk for non-fatal SH (Fig. 1). The detailed results of the regression analyses are shown in the Additional file 1. Lower risk for non-fatal SH was found in those living in institutions at baseline (IRR: 0.23; 95% CI: 0.19–0.29) and those aged 80 and above, The risk for non-fatal SH was slightly higher in those with higher education compared to those with only compulsory education (IRR: 1.18, 95% CI: 1.01–1.37).

### Antidepressant users and non-users

Figure 1 shows that in AD users, age ≥ 80 years was associated with decreased risk for non-fatal SH. An increased risk was found among those with higher education (IRR: 1.34; 95% CI 1.12–1.60).

In non-users of AD, increased risk for non-fatal SH was found in those born in other Nordic countries (IRR: 1.58; 95% CI 1.08–2.29).

### Gender-stratified analyses

#### Men

**Fig. 2:**
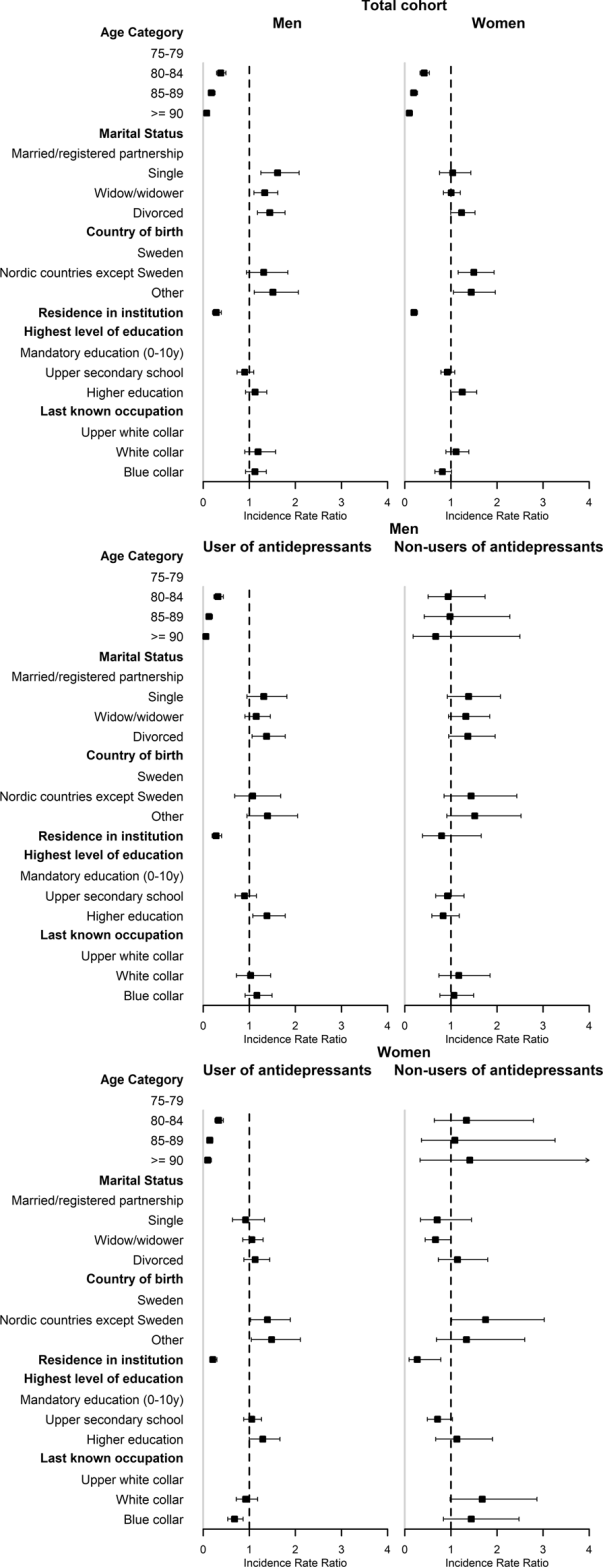
Factors associated with non-fatal self-harm in men and women aged ≥75 years and in antidepressants users and non-users. The regression models were adjusted for age, country of birth, marital status, highest level of education, last registered occupation, monthly individual disposable income, use of specialised psychiatric care, use of other psychoactive medications, residence in institution at baseline and non-fatal self-harm in the previous year

In the total cohort, being unmarried was a risk factor for non-fatal SH (single, IRR: 1.61, 95% CI 1.25–2.08; widowed, IRR: 1.33, 95% CI 1.10–1.62; divorced, IRR: 1.45, 95% CI 1.18–1.78) (Fig. 2 and Additional file 2). A higher risk was found in those born outside of Nordic countries (IRR: 1.51; 95% CI: 1.11–2.07). In AD users, higher risk was found in the divorced (IRR: 1.37; 95% CI: 1.06–1.78).

#### Women

In the total cohort, being foreign-born was associated with increased risk of non-fatal SH. This was the case in those born outside of the Nordic countries (IRR: 1.44; 95% CI: 1.06–1.97), as well as in those from other Nordic countries (IRR: 1.50; 95% CI: 1.16–1.94). In AD users, the risk of non-fatal SH was lower in those who were blue-collar workers before retirement (IRR: 0.68; 95% CI 0.53–0.87).

### Methods of non-fatal SH

**Fig. 3:**
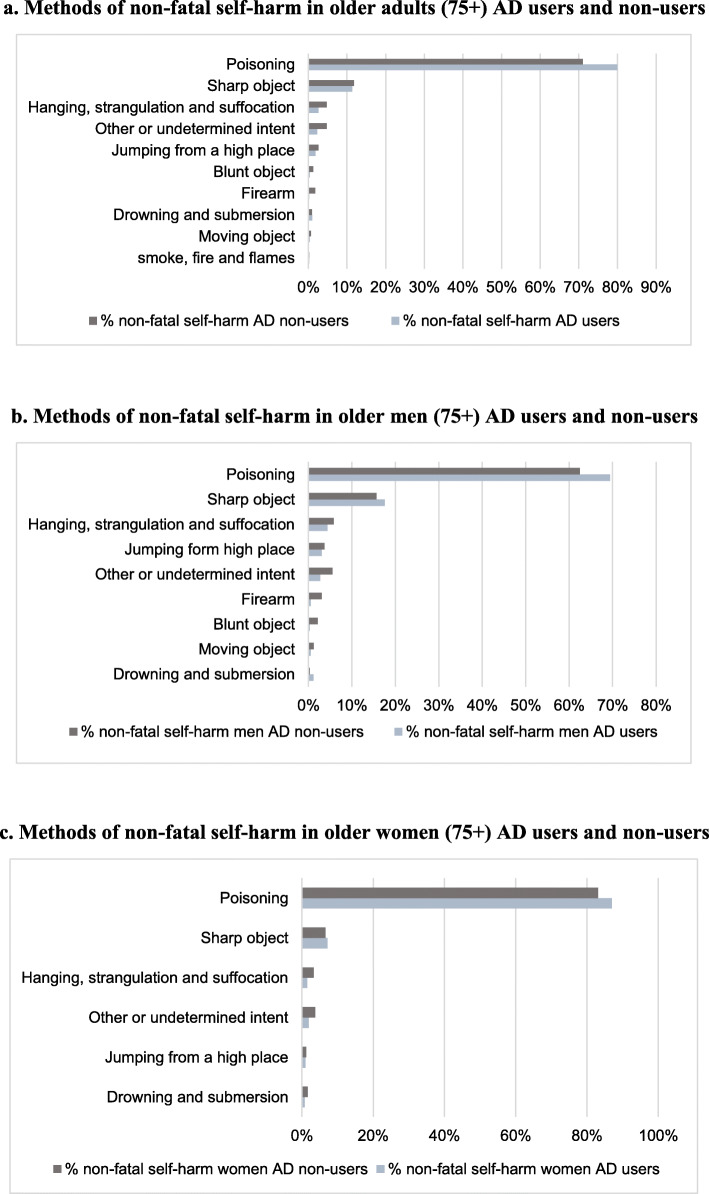
Methods of non-fatal self-harm in all persons aged ≥75 years and in antidepressants users and non-users

Methods of non-fatal SH are shown in Fig. 3. In both genders, poisoning followed by use of sharp objects were the most common methods of non-fatal self-harm. No significant difference in methods of non-fatal SH was observed between users and non-users of AD.

## Discussion

In this national population-based Swedish study, being married was a protective factor for non-fatal SH in men aged 75 years and above, but not in women. Being foreign-born was associated with an increased risk of non-fatal SH. In AD users, an elevated risk of non-fatal SH was found in both men and women with higher education.

The foreign-born older adults seem to constitute a particular risk group for non-fatal SH. This expands on findings from our previous study that focused only on new users of ADs [9]. Taken together, our results point to a need to improve the monitoring of AD treatment and to reach the mental health needs of older adults with foreign background. We have previously shown increased risk of suicidal behaviours in older adults with alcohol use disorders [13], and higher rates of alcohol consumption in other Nordic populations might be a partial explanation of our findings [14]. Old migrants are however not a homogeneous group. Further psychosocial studies should be undertaken to identify factors, which may, under certain conditions, influence seeking for mental healthcare and may imply an increased risk of suicidal behaviour, including the motivation for migration and the distance from host culture.

In the total cohort, risk of non-fatal SH was elevated in those with higher education. This was somewhat unexpected, as we have previously reported an association between lower educational level and non-fatal SH in persons aged 70 and above [15]. Our previous study employed a different methodology, involving a clinical cohort and a population-based comparison group. It is probable that depressed older individuals would be less likely to participate in the latter group, which may have affected results. In the current study, the association between higher education and non-fatal self-harm was particularly pronounced in AD users. Loss of social status might help to explain the risk increase, but it is unclear why we did not see an association in the non-user group.

The lower risk of non-fatal SH in the oldest AD users (80+) may be explained by the extensive use of ADs in institutions and in those with dementia, which are both associated with lower non-fatal SH risk in our cohort. Frailty and severe illnesses in some institutionalized older adults may make them physically or mentally incapable of planning and carrying out a suicidal act.

Our finding of a protective effect of being married on non-fatal SH in older men is in accordance with previous research [16]. The increased risk observed for single and widowed men, in both AD users and non-users, suggests the need to not only focus on men’s use and adherence to prescribed AD therapies but also to recognise and meet the need of social support in older men without partners who are potentially more prone to social isolation. Our study supports the potential value of mental health promotion strategies that encourage men to deconstruct the hegemonic ideal of the man who is emotionally and socially self-sufficient, and to extend their social emotionally supportive relationships other than their partners to achieve this [17].

### Methodological considerations

The use of total population registers provides a more accurate risk estimation as selection bias is eliminated. Further, the large size of the population allowed the inclusion of a wide range of covariates in the adjusted analyses. However, we lacked information on numerous pertinent behavioural risk factors including social isolation, degree of hopelessness, depression severity, problematic alcohol use, and suicidal behaviour earlier in life.

Our study design detected persons who received care in hospital and at specialised outpatient services. The public health significance of studying this group is emphasized by a finding from a British multicentre study estimating that older adult self-harmers presenting to hospital have a risk for suicide that is 67 times that of the general population [18]. However, a limitation of our study is that older adults who self-harmed but did not seek help at hospital or specialised outpatient clinics may have erroneously been considered as controls. This may have caused an underestimation of the risk ratios.

The Swedish Prescribed Drug Register does not include medications prescribed in inpatient settings. As such, there may be a risk that some people classified in this study as non-users of ADs had in fact received this therapy during a hospital stay. We believe this is unlikely to have any noticeable impact on the observed outcome as, in most cases, AD therapy initiated in hospital will be continued after discharge, and thus recorded in the register. The SPDR includes filled prescriptions only, and we are not able to know whether the individuals we identified as users actually took their medications. We note, however, that there is some evidence that older adults taking AD report that the positive aspects of treatment outweigh the negative [19]. The purchase of AD was used as a proxy for depression in our population but we acknowledge a possibility of an indication bias or a residual confounding as AD may be prescribed in late-life for other indications than depression [20].

We lacked data on date of immigration which is a limitation since time spent in the host country may influence the degree of acculturation [21]. Our results may not be extrapolated to other regions or cultures due to differing patterns of suicidal behaviours and availability of healthcare.

## Conclusion

The finding of elevated non-fatal SH risk in persons born outside of Sweden both with and without AD therapies indicates a need for a better understanding of potential barriers to adequate support by mental healthcare service providers, primary care services and social services alike. Considering the risk increase associated with unmarried status in men, there is a special need for interventions that target this group. The complex association between sociodemographic factors and use of antidepressants in the occurrence of self-harm in older men and women highlight the need for more research on tailored multifaceted gender-specific preventive strategies to reduce suicidal behaviour in older adults.

## Supplementary information

**Additional file 1.** Factors associated with non-fatal self-harm in all persons aged ≥75 years and in antidepressants users and non-users.

**Additional file 2.** Factors associated with non-fatal self-harm in men and women aged ≥75 years and in antidepressants users and non-users.

## Data Availability

The datasets generated and/or analysed during the current study are not publicly available due confidentiality as they include sensitive private information. Aggregated data may be available from Statistics Sweden or from the National Board of Health and Welfare upon request.

## Abbreviations

AD: Antidepressants
CI: Confidence interval
IRR: Incidence rate ratio
SH: Self-harm
SPDR: Swedish Prescribed Drug Register
