# Supplementary material for: What are the sociodemographic and gender determinants of non-fatal self-harm in older adult users and non-users of antidepressants? A national population-based study
Source: BMC Public Health. 2020 Jun 16;20:764. doi: 10.1186/s12889-020-08892-2 (PMC7296708; doi:10.1186/s12889-020-08892-2)
Supplement: Supplementary file 1 — Additional file 1. Factors associated with non-fatal self-harm in all persons aged ≥75 years and in antidepressants users and non-users. [file 12889_2020_8892_MOESM1_ESM.rtf]

Online additional material
Table 1. Factors associated with non-fatal self-harm among persons aged 75+, and among antidepressants users and non-users separately*
	All persons aged 75+	Non-users of antidepressants	Users of antidepressants	
Characteristics	N case/control	Crude IRR (95%CI)	Adjusted IRR** (95% CI)	N case/control	Crude IRR (95%CI)	Adjusted IRR** (95% CI)	N
Case/control	Crude IRR (95%CI)	Adjusted IRR** (95% CI)	
Age (years)										
75-79	1127 / 56791	1 (Reference)	1 (Reference)	273 / 13579	1 (Reference)	1 (Reference)	729 / 27764	1 (Reference)	1 (Reference)	
80-84	610 / 29528	1.067 (0.91-1.25)	0.40 (0.33-0.47)	155 / 7625	0.94 (0.63-1.41)	1.07 (0.67-1.72)	412 / 22986	0.32 (0.27-0.38)	0.33 (0.27-0.41)	
85-89	375 / 18295	0.99 (0.79-1.25)	0.18 (0.14-0.24)	104 / 5034	0.82 (0.46-1.46)	1.03 (0.53-2.00)	246 / 16042	0.12 (0.09-0.16)	0.14 (0.10-0.19)	
≥ 90	130 / 7486	0.72 (0.51-1.01)	0.09 (0.06-0.13)	35 / 2112	0.53 (0.25-1.11)	0.95 (0.37-2.48)	89 / 7008	0.05 (0.03-0.08)	0.08 (0.05-0.12)	
Marital status										
Married/Registered partnership	951 / 53428	1 (Reference)	1 (Reference)	271 / 14519	1 (Reference)	1 (Reference)	607 / 30458	1 (Reference)	1 (Reference)	
Single	162 / 7654	1.19 (1.01-1.41)	1.38 (1.13-1.68)	51 / 2175	1.25 (0.92-1.69)	1.18 (0.83-1.67)	98 / 4753	1.03 (0.83-1.28)	1.16 (0.91-1.47)	
Widow/widower	758 / 37419	1.16 (1.04-1.29)	1.15 (1.01-1.31)	155 / 8421	0.97 (0.78-1.21)	0.99 (0.77-1.28)	535 / 28967	0.91 (0.80-1.03)	1.12 (0.96-1.30)	
Divorced	367 / 13180	1.58 (1.39-1.78)	1.37 (1.18-1.59)	87 / 3116	1.49 (1.17-1.91)	1.35 (1.03-1.78)	235 / 9554	1.23 (1.06-1.44)	1.26 (1.06-1.50)	
Country of birth										
Sweden	1950 / 100187	1 (Reference)	1 (Reference)	486 / 25466	1 (Reference)	1 (Reference)	1287 / 66641	1 (Reference)	1 (Reference)	
Other Nordic countries	146 / 5587	1.34 (1.13-1.60)	1.40 (1.14-1.72)	41 / 1293	1.67 (1.20-2.30)	1.58 (1.08-2.29)	90 / 3451	1.35 (1.09-1.68)	1.26 (0.98-1.62)	
Out of Nordic countries 	146 / 6326	1.19 (1.00-1.40)	1.47 (1.18-1.83)	40 / 1591	1.32 (0.95-1.83)	1.44 (0.96-2.15)	99 / 3708	1.39 (1.13-1.71)	1.44 (1.11-1.86)	
Highest level of education										
Mandatory school	1147 / 58838	1 (Reference)	1 (Reference)	311 / 14545	1 (Reference)	1 (Reference)	742 / 40082	1 (Reference)	1 (Reference)	
Secondary school	495 / 25173	1.01 (0.91-1.13)	0.91 (0.80-1.03)	114 / 5809	0.92 (0.74-1.15)	0.81 (0.64-1.04)	332 / 17144	1.05 (0.92-1.20)	0.99 (0.86-1.15)	
Post-secondary or higher	538 / 23332	1.2 (1.08-1.33)	1.18 (1.01-1.37)	114 / 6368	0.84 (0.67-1.05)	0.88 (0.66-1.18)	369 / 14449	1.40 (1.23-1.60)	1.34 (1.12-1.61)	
Last occupation										
Upper white collar worker 	522 / 24910	1 (Reference)	1 (Reference)	115 / 6672	1 (Reference)	1 (Reference)	357 / 15736	1 (Reference)	1 (Reference)	
Lower white collar worker 	346 / 14171	1.18 (1.02-1.35)	1.20 (1.01-1.41)	78 / 3205	1.48 (1.09-1.99)	1.30 (0.94-1.81)	228 / 9783	1.02 (0.86-1.22)	1.04 (0.86-1.27)	
Blue collar worker 	984 / 50894	0.93 (0.83-1.03)	0.96 (0.83-1.11)	263 / 12598	1.23 (0.98-1.54)	1.12 (0.84-1.49)	641 / 34257	0.83 (0.72-0.94)	0.88 (0.74-1.05)	
Monthly individual disposable income										
Q1	478 / 30418	1 (Reference)	1 (Reference)	115 / 7917	1 (Reference)	1 (Reference)	319 / 14835	1 (Reference)	1 (Reference)	
Q2-Q3	1259 / 55970	1.45 (1.30-1.62)	1.02 (0.88-1.17)	331 / 13522	1.72 (1.38-2.14)	1.42 (1.08-1.88)	816 / 39968	0.94 (0.82-1.07)	0.92 (0.77-1.08)	
Q4	505 / 25712	1.28 (1.12-1.46)	0.78 (0.65- 0.94)	121 / 6911	1.24 (0.95-1.63)	1.07 (0.76-1.52)	341 / 18997	0.82 (0.69-0.96)	0.69 (0.56-0.86)	
Residence in institution	120 / 8697	0.66 (0.55-0.80)	0.23 (0.19-0.29)	19 / 909	1.05 (0.66-1.68)	0.52 (0.28-0.94)	93 / 15151	0.24 (0.20-0.30)	0.24 (0.19- 0.31)	
Previous non-fatal self-harm	145 / 148	55.42 (43.53-70.56)	19.69 (14.03-27.63)	9 / 13	36.23 (15.23-86.20)	29.97 (9.11-98.59)	124 / 268	25.34 (20.31-31.61)	17.01(12.78-22.63)	
*Since nested case-control matching is stratified by gender the crude IRR=1 and not applicable in adjusted model
**Adjusted for age, country of birth, marital status, highest level of education, last registered occupation, monthly individual disposable income, use of specialised psychiatric care, use of other psychoactive medications, nursing home residence and non-fatal self-harm in the previous year.
IRR: Incidence rate ratio; Q: Quartile
