# Supplementary material for: What are the sociodemographic and gender determinants of non-fatal self-harm in older adult users and non-users of antidepressants? A national population-based study
Source: BMC Public Health. 2020 Jun 16;20:764. doi: 10.1186/s12889-020-08892-2 (PMC7296708; doi:10.1186/s12889-020-08892-2)
Supplement: Supplementary file 2 — Additional file 2. Factors associated with non-fatal self-harm in men and women aged ≥75 years and in antidepressants users and non-users. [file 12889_2020_8892_MOESM2_ESM.rtf]

Online additional material 2. Factors associated with non-fatal self-harm among men and women in the total cohort and among antidepressants users and non-users separately*
Table 1. Factors associated with non-fatal self-harm among men in the total cohort and among antidepressants users and non-users separately*
	All men aged 75+	Men non-users of antidepressants	Men users of antidepressants	
Characteristics	N case/control	Crude IRR
(95%CI)	Adjusted IRR** (95% CI)	N case/control	Crude IRR (95%CI)	Adjusted IRR** (95% CI)	N
Case/control	Crude IRR (95%CI)	Adjusted IRR** (95% CI)	
Age (years)										
75-79	503 / 25496	1 (Reference)	1 (Reference)	167 / 8201	1 (Reference)	1 (Reference)	297 / 11420	1 (Reference)	1 (Reference)	
80-84	251 / 12318	1.11 (0.87-1.43)	0.38 (0.29-0.49)	81 / 4177	0.80 (0.46-1.38)	0.94 (0.51-1.74)	157 / 8853	0.32 (0.24-0.43)	0.32 (0.24-0.44)	
85-89	169 / 7961	1.18 (0.83-1.68)	0.18 (0.12-0.26)	61 / 2864	0.80 (0.37-1.71)	0.98 (0.42-2.28)	99 / 6225	0.12 (0.08-0.19)	0.12 (0.08-0.20)	
≥ 90	57 / 3225	0.88 (0.52-1.47)	0.08 (0.04-0.14)	16 / 1008	0.46 (0.16-1.31)	0.67 (0.18-2.50)	39 / 3102	0.05 (0.03-0.09)	0.06 (0.03-0.12)	
Marital status										
Married/Registered partnership	524 / 30797	1 (Reference)	1 (Reference)	178 / 10329	1 (Reference)	1 (Reference)	321 / 17011	1 (Reference)	1 (Reference)	
Single	99 / 4086	1.43 (1.15-1.77)	1.61 (1.25-2.08)	37 / 1409	1.52 (1.07-2.18)	1.38 (0.92-2.08)	56 / 2402	1.24 (0.93-1.65)	1.31 (0.96- 1.82)	
Widow/widower	203 / 8550	1.42 (1.19-1.68)	1.33 (1.10-1.62)	62 / 2766	1.31 (0.97-1.78)	1.32 (0.95-1.845)	127 / 6597	1.01 (0.82-1.25)	1.15 (0.91- 1.46)	
Divorced	151 / 5381	1.65 (1.37-1.98)	1.45 (1.18-1.78)	45 / 1671	1.56 (1.12-2.16)	1.37 (0.95-1.96)	88 / 3560	1.32 (1.03-1.67)	1.38 (1.06-1.78)	
Country of birth										
Sweden	867 / 44447	1 (Reference)	1 (Reference)	284 / 14739	1 (Reference)	1 (Reference)	524 / 27046	1 (Reference)	1 (Reference)	
Other Nordic countries	51 / 1940	1.35 (1.01-1.80)	1.31 (0.94-1.84)	21 / 638	1.71 (1.09-2.68)	1.44 (0.85-2.43)	27 / 1098	1.27 (0.86-1.89)	1.07 (0.68-1.68)	
Out of Nordic countries 	62 / 2613	1.22 (0.94-1.58)	1.51 (1.11-2.07)	20 / 873	1.19 (0.75-1.89)	1.52 (0.91-2.52)	41 / 1456	1.46 (1.06-2.01)	1.40 (0.95-2.05)	
Highest level of education										
Mandatory school	501 / 24282	1 (Reference)	1 (Reference)	177 / 8048	1 (Reference)	1 (Reference)	292 / 15002	1 (Reference)	1 (Reference)	
Secondary school	164 / 8945	0.89 (0.75-1.07)	0.90 (0.74-1.10)	57 / 2798	0.93 (0.69-1.26)	0.93 (0.67-1.29)	97 / 5558	0.90 (0.72-1.14)	0.90 (0.70-1.15)	
Post-secondary or higher	288 / 13852	1.01 (0.87-1.17)	1.13 (0.92-1.38)	76 / 4597	0.75 (0.57-0.99)	0.83 (0.59-1.18)	191 / 8218	1.20 (1.00-1.44)	1.38 (1.08-1.78)	
Last occupation										
Upper white collar worker 	280 / 14750	1 (Reference)	1 (Reference)	83 / 4739	1 (Reference)	1 (Reference)	172 / 8864	1 (Reference)	1 (Reference)	
Lower white collar worker 	85 / 3598	1.24 (0.97-1.58)	1.19 (0.90-1.57)	30 / 1153	1.44 (0.94-2.21)	1.17 (0.74-1.85)	47 / 2215	1.09 (0.78-1.51)	1.03 (0.72-1.47)	
Blue collar worker 	476 / 21657	1.16 (1.00-1.35)	1.12 (0.92-1.37)	165 / 7146	1.31 (1.00-1.72)	1.07 (0.76-1.50)	286 / 13294	1.11 (0.92-1.35)	1.17 (0.91-1.50)	
Monthly individual disposable income										
Q1	96 / 7429	1 (Reference)	1 (Reference)	40 / 3007	1 (Reference)	1 (Reference)	51 / 2396	1 (Reference)	1 (Reference)	
Q2-Q3	567 / 24774	1.79 (1.44-2.23)	1.12 (0.86-1.47)	197 / 7926	1.90 (1.34-2.69)	1.52 (0.98-2.35)	335 / 15364	1.02 (0.76-1.38)	0.89 (0.62-1.27)	
Q4	317 / 16797	1.48 (1.17-1.87)	0.88 (0.65-1.19)	88 / 5317	1.27 (0.86-1.86)	1.08 (0.66-1.76)	206 / 11840	0.81 (0.59-1.11)	0.70 (0.47-1.03)	
Residence in institution	58 / 3039	0.95 (0.72-1.25)	0.28 (0.20-0.39)	13 / 406	1.66 (0.93-2.95)	0.80 (0.39-1.66)	44 / 5973	0.31 (0.22-0.42)	0.28 (0.20-0.40)	
Previous non-fatal self-harm	66 / 63	62.12 (42.83-90.10)	21.47 (13.07-35.27)	6 / 9	33.33 (11.87-93.65)	27.50 (6.84- 110.55)	53 / 124	23.20 (16.63-32.36)	19.35 (12.68-29.53)	
*Since nested case-control matching is stratified by gender the crude IRR=1 and not applicable in adjusted model
**Adjusted for age, country of birth, marital status, highest level of education, last registered occupation, monthly individual disposable income, use of specialised psychiatric care, use of other psychoactive medications, residence in institution and non-fatal self-harm in the previous year.
IRR: Incidence rate ratio; Q: Quartile

Table 2. Factors associated with non-fatal self-harm among women in the total cohort and among antidepressants users and non-users separately*

	All women aged 75+	Women non-users of antidepressants	Women users of antidepressants	
Characteristics	N case/control	Crude IRR (95%CI)	Adjusted IRR** (95% CI)	N case/control	Crude IRR (95%CI)	Adjusted IRR** (95% CI)	N
Case/control	Crude IRR (95%CI)	Adjusted IRR** (95% CI)	
Age (years)										
75-79	624 / 31295	1 (Reference)	1 (Reference)	106 / 5378	1 (Reference)	1 (Reference)	432 / 16344	1 (Reference)	1 (Reference)	
80-84	359 / 17210	1.04 (0.84-1.28)	0.42 (0.33- 0.53)	74 / 3448	1.16 (0.63-2.15)	1.34 (0.64-2.79)	255 / 14133	0.32 (0.25-0.40)	0.33 (0.26-0.44)	
85-89	206 / 10334	0.86 (0.64-1.19)	0.19 (0.13-0.28)	43 / 2170	0.82 (0.33-2.00)	1.09 (0.36-3.26)	147 / 9817	0.12 (0.09-0.17)	0.14 (0.10-0.21)	
≥ 90	73 / 4261	0.63 (0.40-0.98)	0.10 (0.06-0.17)	19 / 1104	0.58 (0.19-1.75)	1.41 (0.33-5.98)	50 / 3906	0.05 (0.03-0.09)	0.10 (0.05-0.18)	
Marital status										
Married/Registered partnership	427 / 22631	1 (Reference)	1 (Reference)	93 / 4190	1 (Reference)	1 (Reference)	286 / 13447	1 (Reference)	1 (Reference)	
Single	63 / 3568	0.93 (0.71-1.22)	1.04 (0.76-1.44)	14 / 766	0.79 (0.44-1.40)	0.70 (0.34-1.45)	42 / 2351	0.83 (0.60-1.15)	0.92 (0.64-1.33)	
Widow/widower	555 / 28869	1.01 (0.88-1.16)	1.01 (0.84-1.21)	93 / 5655	0.70 (0.51-0.95)	0.67 (0.44-1.00)	408 / 22370	0.84 (0.72-0.99)	1.06 (0.86-1.30)	
Divorced	216 / 7799	1.47 (1.24-1.73)	1.23 (0.10-1.52)	42 / 1445	1.30 (0.89-1.88)	1.14 (0.73-1.80)	147 / 5994	1.15 (0.94-1.41)	1.13 (0.88-1.44)	
Country of birth										
Sweden	1083 / 55740	1 (Reference)	1 (Reference)	202 / 10727	1 (Reference)	1 (Reference)	763 / 39595	1 (Reference)	1 (Reference)	
Other Nordic countries	95 / 3647	1.34 (1.08-1.66)	1.50 (1.16-1.94)	20 / 655	1.63 (1.02-2.59)	1.75 (1.02-3.02)	63 / 2353	1.39 (1.07-1.81)	1.39 (1.03- 1.89)	
Out of Nordic countries 	84 / 3713	1.17 (0.93-1.46)	1.44 (1.06-1.97)	20 / 718	1.48 (0.93-2.36)	1.34 (0.69-2.60)	58 / 2252	1.34 (1.02-1.76)	1.48 (1.04-2.11)	
Highest level of education										
Mandatory school	646 / 34556	1 (Reference)	1 (Reference)	134 / 6497	1 (Reference)	1 (Reference)	450 / 25080	1 (Reference)	1 (Reference)	
Secondary school	331 / 16228	1.11 (0.97-1.27)	0.92 (0.79-1.08)	57 / 3011	0.93 (0.68-1.27)	0.71 (0.49-1.03)	235 / 11586	1.14 (0.97-1.34)	1.06 (0.88-1.27)	
Post-secondary or higher	250 / 9480	1.44 (1.24-1.67)	1.25 (0.99-1.56)	38 / 1771	1.05 (0.73-1.52)	1.13 (0.67-1.90)	178 / 6231	1.62 (1.35-1.93)	1.29 (1.01-1.67)	
Last occupation										
Upper white collar worker 	242 / 10160	1 (Reference)	1 (Reference)	32 / 1933	1 (Reference)	1 (Reference)	185 / 6872	1 (Reference)	1 (Reference)	
Lower white collar worker 	261 / 10573	1.02 (0.85-1.22)	1.11 (0.89-1.39)	48 / 2052	1.42 (0.90-2.23)	1.68 (0.98-2.87)	181 / 7568	0.88 (0.71-1.08)	0.92 (0.72-1.18)	
Blue collar worker 	508 / 29237	0.72 (0.62-0.84)	0.82 (0.66-1.01)	98 / 5452	1.08 (0.72-1.62)	1.44 (0.84-2.48)	355 / 20963	0.62 (0.52- 0.75)	0.68 (0.53-0.87)	
Monthly individual disposable income										
Q1	382 / 22989	1 (Reference)	1 (Reference)	75 / 4910	1 (Reference)	1 (Reference)	268 / 12439	1 (Reference)	1 (Reference)	
Q2-Q3	692 / 31196	1.34 (1.18-1.52)	1.03 (0.86-1.23)	134 / 5596	1.58 (1.18-2.10)	1.59 (1.07-2.37)	481 / 24604	0.91 (0.78-1.06)	0.92 (0.75-1.13)	
Q4	188 / 8915	1.28 (1.07-1.52)	0.82 (0.64-1.06)	33 / 1594	1.37 (0.90-2.09)	1.54 (0.88-2.70)	135 / 7157	0.88 (0.71-1.08)	0.71 (0.54-0.95)	
Residence in institution	62 / 5658	0.51 (0.39-0.66)	0.20 (0.14- 0.28)	6 / 503	0.58 (0.25-1.32)	0.27 (0.09-0.78)	49 / 9178	0.21 (0.15-0.28)	0.21 (0.14- 0.30)	
Previous non-fatal self-harm	79 / 85	50.94 (37.05-70.03)	18.01 (11.30-28.69)	3 / 4	44.52 (8.86-223.59)	50.95 (5.49-472.97)	71 / 144	27.22 (20.23-36.625)	15.48 (10.50-22.81)	
*Since nested case-control matching is stratified by gender the crude IRR=1 and not applicable in adjusted model
**Adjusted for age, country of birth, marital status, highest level of education, last registered occupation, monthly individual disposable income, use of specialised psychiatric care, use of other psychoactive medications, residence in institution and non-fatal self-harm in the previous year.
IRR: Incidence rate ratio; Q: Quartile
